# Differential inequalities imposed by the extended hypergeometric function

**DOI:** 10.1186/s40064-016-1996-9

**Published:** 2016-03-25

**Authors:** Rabha W. Ibrahim, M. Z. Ahmad, Hiba F. Al-Janaby

**Affiliations:** Faculty of Computer Science and Information Technology, University Malaya, 50603 Kuala Lumpur, Malaysia; Institute of Engineering Mathematics, Universiti Malaysia Perlis, 02600 Arau, Malaysia

**Keywords:** Analytic function, Univalent function, Integral operator, Subordination, Superordination, Hypergeometric function, Unit disk, 30C45

## Abstract

Recently, the generalized hypergeometric function is extended by utilizing the Beta function. Based on this type of function, we introduce a new operator in the open unit disk. The present article investigates some subordination and superordination results for certain normalized analytic functions in the open unit disk, which are acted upon by the generalized Noor integral operator. Some of outcomes improve and generalize previously known outcomes.

## Background

The theory of hypergeometric functions theaters significant and imposing role in the study of the fractional calculus and the geometric function theory. It motivates the theory of univalent functions, by attractive to current research after their utilization in the proof of great famous problem in geometric function theory which is called by the Bieberbach’s conjecture. This theory has been developed with enriched many presentations and simplification by protruding complex analysis.

Let $H(\mathcal{U})$ be the class of all holomorphic functions $\phi (z)$ which are defined in the unit disk $\mathcal{U}$. For $\alpha \in \mathbb {C}$ and $n\in \mathbb {N}$, we let $H \left[ a,n\right] =\{ {\phi \in H(\mathcal{U}):\phi (z)=a+a_{n} z^{n} +a_{n+1} z^{n+1}+\cdots }\}$ and $\mathcal A$ be the subclass of $H(\mathcal{U})$ consisting of functions of the form

1 $$\phi (z) = z + \sum _{n = 2}^\infty {a_n z^n},\quad (z\in \mathcal{U}).$$

For functions $\phi (z)$, given (1), and $\psi (z)$ given by

2 $$\psi (z) = z + \sum _{n = 2}^\infty {b_n z^n},\quad (z \in \mathcal{U}).$$

the Hadamard product (or convolution) of $\phi (z)$ and $\psi (z)$ is defined by

3 $$(\phi *\psi )(z) = z + \sum _{n = 2}^\infty {a_n b_n z^n},\quad (z \in \mathcal{U}).$$

For $\phi$ and $\varphi$ be members of the function class $H(\mathcal{U})$, the function $\phi$ is said to be subordinate to $\varphi$, or $\varphi$ is superordinate to $\phi$, if there is an analytic function $\theta (z)$ in $\mathcal{U}$ with $\theta (0)=0$ and $|\theta (z)|<1$ for all $z\in \mathcal{U}$, such that $\phi (z)={\varphi {(\theta (z))}}$. In this case, we write $\phi \prec \varphi ,$ or $\phi (z)\prec \varphi (z).$ Furthermore, if the function $\varphi$ is univalent in $\mathcal{U},$ then we have the following equivalence (Miller and Mocanu 2000):

$$\phi (z)\prec \varphi (z)\quad (z\in \mathcal{U}) \iff \phi (0)=\varphi (0),\quad\phi (\mathcal{U})\subset \varphi (\mathcal{U}).$$

Let $\phi :\mathbb {C}^2\longrightarrow \mathbb {C}$ and let $\vartheta$ be univalent in $z\in \mathcal{U}$. If $\rho$ is analytic in $\mathcal{U}$ and satisfies the differential subordination $\phi (\rho (z),z\rho ^{\prime }(z))\prec \vartheta (z)$ then $\rho$ is called a solution of the differential subordination. The univalent function $\eta$ is called a dominant of the solutions of the differential subordination, $\rho \prec \eta$. If $\rho$ and $\phi (\rho (z),z\rho ^{\prime }(z))$ are univalent in $\mathcal{U}$ and satisfy the differential superordination $\vartheta (z) \prec \phi (\rho (z),z\rho ^{\prime }(z))$ then $\rho$ is called a solution of the differential superordination. An analytic $\eta$ is called subordinate of the solution of the differential superordination if $\eta \prec \rho$ (Miller and Mocanu 2003).

For real or complex numbers $\alpha ,\beta ,\gamma$ other than $0,-1,-2,\ldots$ the Gaussian hypergeometric function is defined by de Branges (1985)

4 $$F(\alpha ,\beta ;\gamma ,z)=\sum _{n=0}^{\infty }\frac{(\alpha )_n\,(\beta )_n}{(\gamma )_n\,(1)_n}\,z^n =1+\frac{\alpha \beta }{\gamma }z+\frac{\alpha (\alpha +1)\beta (\beta +1)}{\gamma (\gamma +1)}\frac{z^2}{2!}+\cdots$$

where $(\alpha )_n$ is the Pochhammer symbol defined by

$$(\alpha )_n=\frac{\Gamma (\alpha +n)}{\Gamma (\alpha )}=\left\{ \begin{array}{ll} 1,&\quad n=0\\ \alpha (\alpha +1)\ldots(\alpha +n-1), &\quad n=\{1,2,\ldots \} \end{array}\right.$$

and achieved

$$\begin{aligned}&(i)\,\,\, n(\wp )_n=\wp \left[ (\wp +1)_n-(\wp )_n\right] \\&(ii)\,\,\,(\wp )_{n+1}=\wp (\wp +1)_n=(\wp +n)(\wp )_n. \end{aligned}$$

Let *B*(*x*, *y*) be the familiar Beta function defined by Srivastava et al. (2012, p. 8))

$$B(x,y)=\left\{ \begin{array}{ll} \int _{0}^{1}\sigma ^{x-1}(1-\sigma )^{y-1}d\sigma &\quad (\mathfrak {R}(x)>0;\mathfrak {R}(y)>0)\\ \frac{\Gamma (x)\Gamma (y)}{\Gamma (x+y)}&\quad (x,y \in \mathbb {C}\,\mathbb {Z}^-_0) \end{array}\right.$$

Here $\Gamma$ denotes the Euler’s Gamma function (see, e.g., Srivastava and Choi 2012, Section 1.1). Srivastava et al. (2014) $\mathcal{B}^{a,b;\kappa ,\mu }_p(x,y),$ introduced the extended Beta function as follows

5 $$\begin{aligned}&\mathcal{B}^{a,b;\kappa ,\mu }_p(x,y)=\int _{0}^{1}\sigma ^{x-1}(1-\sigma )^{y-1} F\left( a;b;-\frac{p}{\sigma ^{\kappa }(1-\sigma )^{\mu }}\right) d\sigma , \\&\quad\left( \kappa \ge 0, \mu \ge 0,\,\mathfrak {R}(p)\ge 0,\,\min \{\mathfrak {R}(a),\mathfrak {R}(b)\}>0, \mathfrak {R}(x)>-\mathfrak {R}(\kappa a), \mathfrak {R}(y)>-\mathfrak {R}(\mu a)\right) . \end{aligned}$$

The special case of (5) when $p=0$ is seen to immediately reduce to the familiar beta function *B*(*x*, *y*) (min $\{\mathfrak {R}(x), \mathfrak {R}(y)\}>0$) (Srivastava and Choi 2012).

Agarwal et al. (2015) introduced the extended Gauss hypergeometric function as follows

6 $$\begin{aligned}&F_{p;\kappa ,\mu }(\alpha ,\beta ;\gamma ;z;m):= \sum _{n=0}^{\infty }\frac{(\alpha )_n(\beta )_n}{(\gamma )_n}\frac{B^{a,b;\kappa , \mu }_p(\beta +n,\gamma -\beta +m)}{B(\beta +n,\gamma -\beta +m)}\frac{z^n}{n!} \\&\quad\left( p\ge 0 ,\, \mathfrak {R}(\kappa )>0, \, \mathfrak {R}(\mu )>0, \, m<\mathfrak {R}(\beta )< \mathfrak {R}(\gamma ), \,|z|<1\right) . \end{aligned}$$

The special case of (6) $p=0,\,m=0$ is noted to reduce to the ordinary Gauss hypergeometric function $F(\alpha ,\beta ;\gamma ;z)$ (Agarwal et al. 2015). Ibrahim et al. (2015b) introduced a generalized Noor integral operator using a fractional hypergeometric function as follows:

7 $$\begin{aligned}&Q^{\wp }_{p;\kappa ,\mu }(\gamma ;\alpha ,\beta ;z;m):\mathcal{A}\longrightarrow \mathcal{A}, \\&Q^{\wp }_{p;\kappa ,\mu }(\gamma ;\alpha ,\beta ;z;m)\phi (z)= \Omega \left( zF^{a,b;\kappa ,\mu }_p(\alpha , \beta ;\gamma ;z;m)\right) ^{-1}*\phi (z),\,\,\,(z\in \mathcal{U}) \\&Q^{\wp }_{p;\kappa ,\mu }(\gamma ;\alpha ,\beta ;z;m)\phi (z)=z+\sum _{n=2}^{\infty } \frac{(\gamma )_{n-1}}{(\alpha )_{n-1}(\beta )_{n-1}}\frac{\Omega \,B(\beta +n-1,\gamma -\beta +m)}{B^{a,b;\kappa ,\mu }_p(\beta +n-1,\gamma -\beta +m)}(\wp +1)_{n-1}\,a_n\,z^n, \end{aligned}$$

where

$$\Omega =\frac{B^{a,b;\kappa ,\mu }_p(\beta ,\gamma -\beta +m)}{B(\beta ,\gamma -\beta +m)},$$

$$\left( zF^{a,b;\kappa ,\mu }_p(\alpha ,\beta ;\gamma ;z;m)\right) ^{-1}= \sum _{n=1}^{\infty }\frac{(\gamma )_{n-1}}{(\alpha )_{n-1}(\beta )_{n-1}}\frac{B(\beta +n-1,\gamma -\beta +m)}{B^{a,b;\kappa ,\mu }_p(\beta +n-1,\gamma -\beta +m)}(\wp +1)_{n-1}\,z^n.$$

In view of (7), we get

8 $$z\left[ Q^{\wp }_{p;\kappa ,\mu }(\gamma ;\alpha ,\beta ;z;m)\phi (z)\right] ^{\prime }=(\wp +1) \,Q^{\wp +1}_{p;\kappa ,\mu }(\gamma ;\alpha ,\beta ;z;m)\phi (z)-\wp \,Q^{\wp }_{p;\kappa ,\mu }(\gamma ;\alpha ,\beta ;z;m)\phi (z).$$

In particular, we have

$$Q^{\wp }_{0;\kappa ,\mu }(\gamma ;\alpha ,\beta ;z;0)\phi (z)= I_\wp (\alpha ,\beta ,\gamma )\phi (z)$$

where the integral operator $I_\wp (\alpha ,\beta ,\gamma )\phi (z)$ was investigate by Noor (2006).

Making use of the principle of subordination various subordination theorems involving certain operators for analytic functions in $\mathcal{U}$ were investigated by Miller and Mocanu (2000) and Owa and Srivastava (2004). Further, Bulboaca (2002a, b) and Miller and Mocanu (2003) extended the study to differential superordination as the dual problem of differential subordination, later the study has been taken by many researchers such as, Ali et al. (2005), Murugusundaramoorthy and Magesh (2006), and Shanmugam et al. (2006), Magesh and Murugusundaramoorthy (2008), Mostafa and Aouf (2009), Aouf and Mostafa (2010), Cho et al. (2011), Magesh (2011), Ibrahim et al. (2015a), and others.

Related to the present investigation, we mention some of them in recent years. In Ibrahim and Darus (2008), the first author applied a method based on the differential subordination and superordination in order to obtain results involving generalized Noor integral operator utilizing the Fox- Wright function for a normalized analytic function $\phi (z),\, z\in \mathcal{U}$ and is denoted by $I_\lambda [(\alpha _j,A_j)_{1,q};(\beta _j,B_j)_{1,p}]\phi (z)$. Also they studied the sufficient condition to satisfy

$$\eta _1(z)\prec \frac{[zI_\lambda [(\alpha _j, A_j)_{1,q};(\beta _j,B_j)_{1,p}]\phi (z)]'}{\Phi [I_\lambda [(\alpha _j, A_j)_{1,q};(\beta _j,B_j)_{1,p}]\phi (z)]}\prec \eta _2(z),$$

for some convex functions $\eta _1$ and $\eta _2$. with $\eta _1(0)\ne 0$ and $\eta _2(0)\ne 0$.

Aouf and Seoudy (2013) investigated some subordination and superordination results for certain p-valent functions in the open unit disc, which acted upon by a class of a linear operator denoted by $I^{m,l}_{p,q,s,\lambda }(\alpha _1)\phi (z)$. Further, they studied the sufficient condition to satisfy

$$\eta _1(z)\prec \left[ \frac{I^{m,l}_{p,q,s,\lambda }(\alpha _1)\phi (z)}{z^p}\right] ^{\mu }\left[ \frac{z^p}{I^{m,l}_{p,q,s,\lambda }(\alpha _1+1)\phi (z)}\right] ^{\nu }\prec \eta _2(z),$$

for some convex functions $\eta _1$ and $\eta _2$. with $\eta _1(0)\ne 0$ and $\eta _2(0)\ne 0$.

Magesh et al. (2014) studied the subordination and superordination results of the linear operator denoted by $\Theta [\alpha _1](\phi )(z)$. Also, they discussed the sufficient condition to satisfy

$$\eta _1(z)\prec \left[ \frac{\Theta [\alpha _1](\phi *\Phi )(z)}{z}\right] ^{\mu }\left[ \frac{z}{\Theta [\alpha _1+1](\phi *\Psi (z))}\right] ^{\nu }\prec \eta _2(z),$$

for some convex functions $\eta _1$ and $\eta _2$ with $\eta _1(0)\ne 0$ and $\eta _2(0)\ne 0$ and $\Phi (z) = z + \sum _{n = 2}^\infty {\lambda _n z^n}, \Psi (z) = z + \sum _{n = 2}^\infty {\mu _n z^n}$ are analytic functions in $\mathcal{U}$ with $\lambda _n\ge 0, \mu _n\ge 0\,\, \text{ and }\,\, \lambda _n\ge \mu _n.$

Ibrahim et al. (2015b) investigated some differential subordination and superordination results regarding the generalized integral operator defined by (7). Moreover, we investigate sufficient condition for a normalized analytic function $\phi (z),z\in \mathcal{U}$ to satisfy

$$\eta _1(z)\prec \frac{z\left[ Q^{\wp }_{p;\kappa ,\mu } (\gamma ;\alpha ,\beta ;z;m)\phi (z)\right] '}{\Phi \left[ Q^{\wp }_{p;\kappa ,\mu }(\gamma ;\alpha ,\beta ;z;m)\phi (z)\right] }\prec \eta _2(z),$$

for some convex functions $\eta _1$ and $\eta _2$.

In this present paper, we study some differential subordination and superordination results for new subclasses regarding the generalized integral operator defined by (7). Moreover, we investigate sandwich results containing the given generalized integral operator for certain a normalized analytic function $\phi (z),z\in \mathcal{U}$ such that $(\phi *\Theta )(z)\ne 0$ to satisfy

$$\eta _1(z)\prec \left[ \frac{Q^{\wp }_{p;\kappa ,\mu }(\gamma ;\alpha ,\beta ;z;m) (\phi *\Phi )(z)}{z}\right] ^{\sigma }\left[ \frac{z}{Q^{\wp }_{p;\kappa ,\mu } (\gamma ;\alpha ,\beta ;z;m)(\phi *\Theta )(z)}\right] ^{\nu }\prec \eta _2(z),$$

for some convex functions $\eta _1$ and $\eta _2$ and $\Phi (z) = z + \sum _{n = 2}^\infty {\lambda _n z^n}, \Psi (z) = z + \sum _{n = 2}^\infty {\mu _n z^n}$ are analytic functions in $\mathcal{U}$ with $\lambda _n\ge 0,\mu _n\ge 0\,\, \text{ and }\,\, \lambda _n\ge \mu _n$.

## Preliminaries

In order to prove our subordination and superordination results, we need to the following lemmas in the sequel.

### **Definition 1**

(Miller and Mocanu 2003) Let $\mathcal Q$ denote the set of functions $\phi$ that are analytic and univalent on $\overline{ \mathcal{U}}\backslash { \mathcal{E}(\phi )}$, where ${\mathcal{E}(\phi )}=\{\xi \in \partial \mathcal{U} :\lim \limits _{z\longrightarrow \xi }\phi (z)=\infty \}$, is such that $\min |\phi '(\xi )|=p >0$ for $\xi \in \partial { \mathcal{U}} \backslash {\mathcal{E}({\phi })}$.

### **Lemma 1**

(Miller and Mocanu 2000) *Let*$\rho$*be univalent in the unit disk*$\mathcal{U}$*and*$\Psi$*and let*$\Lambda$*be analytic in a domain*$\mathcal{D}$*containing*$\rho (\mathcal{U})\,\,\text{ with }\,\,\phi (\omega )\ne 0$*when*$\omega \in \eta (\mathcal{U})$. *Set*$\mathcal{Q}(z):=z\eta ^{\prime }(z) \Lambda \left( \eta (z)\right) \,\,\text{ and }\,\,\vartheta (z) :=\Psi \left( \rho (z)\right) +\mathcal{Q}(z)$. *Suppose that*

1. $\mathcal{Q}(z)$*is starlike univalent in*$\mathcal{U}$,
2. $$\mathfrak {R}(z\vartheta ^{\prime }(z)/\mathcal{Q}(z))>0$$*for*$$z \in \mathcal{U}$$.

*If*$\rho$*is analytic with*$\rho (0)=\eta (0),\,\rho (\mathcal{U})\subset \mathcal{D}$*and*

$$\Psi \left( \rho (z)\right) +z\rho ^{\prime }(z)\Lambda \left( \rho (z)\right) \prec \Psi \left( \eta (z)\right) +z\eta ^{\prime }(z)\Lambda \left( \eta (z)\right) ,$$

*then*$\rho (z)\prec \eta (z)$, *and*$\eta (z)$*is the best dominant*.

### **Lemma 2**

(Bulboaca 2002a) *Let*$\eta$*be convex univalent in the unit disk*$\mathcal{U}$*and*$\Pi$*and let*$\Delta$*be analytic in a domain*$\mathcal{D}$*containing*$\eta (\mathcal{U})$. *Suppose that*

1. $z\eta ^{\prime }(z)\Delta \left( \eta (z)\right)$*is starlike univalent in*$\mathcal{U}$,
2. $$\mathfrak {R}\{\Pi '\left( \eta (z)\right) /\Delta (\eta (z))\})>0$$*for*$$z\in \mathcal{U}$$.

*If*$\rho (z)\in H[\eta (0),1]\cap \mathcal{Q}$*with*$\rho (\mathcal{U})\subseteq \mathcal{D}$*and*$\Pi \left( \rho (z)\right) +z\rho ^{\prime }(z)\Delta \left( \rho (z)\right)$*being univalent in*$\mathcal{U}$*and*

$$\Pi \left( \eta (z)\right) +z\eta ^{\prime }(z)\Delta \left( \eta (z)\right) \prec \Pi \left( \rho (z)\right) +z\rho ^{\prime }(z)\Delta \left( \rho (z)\right) ,$$

*then*$\eta (z)\prec \rho (z)$, *and*$\eta (z)$*is the best subordinant*.

## Sandwich outcomes

By making use of Lemmas 1, we first prove the following subordination results.

### **Theorem 1**

*Let*$\Phi ,\Psi \in \mathcal{A},\,\lambda _i\in \mathbb {C}\,(i=1,2,3),\,\lambda _3\ne 0,\,\sigma ,\,\nu \in \mathbb {C}$*and*$\eta (z)\ne 0$*be univalent in*$\mathcal{U}$*such that*$z\eta ^{\prime }(z)/\eta (z)$*is starlike univalent in*$\mathcal{U}$*and*

9 $$\mathfrak {R}\left\{ 1+\frac{\lambda _2}{\lambda _3}\,\eta (z)+\frac{z \eta ^{\prime \prime }(z)}{\eta ^{\prime }(z)}-\frac{z\eta ^{\prime }(z)}{\eta (z)}\right\} > 0, \quad (z \in \mathcal{U}).$$

*If*$\phi \in \mathcal{A}$*satisfies the subordination*

$$\begin{aligned} &\lambda _1+\lambda _2\,\left[ \frac{Q^{\wp }_{p;\kappa ,\mu }(\gamma ;\alpha ,\beta ;z;m) (\phi *\Phi )(z)}{z}\right] ^{\sigma } \left[ \frac{z}{Q^{\wp }_{p;\kappa ,\mu }(\gamma ;\alpha ,\beta ;z;m) (\phi *\Theta )(z)}\right] ^{\nu }\\&\quad+\lambda _3\sigma (\wp +1)\left[ \frac{Q^{\wp +1}_{p;\kappa ,\mu }(\gamma ;\alpha ,\beta ;z;m)(\phi *\Phi )(z)}{Q^{\wp }_{p;\kappa ,\mu } (\gamma ;\alpha ,\beta ;z;m)(\phi *\Phi )(z)}-1\right] +\nu (\wp +2)\left[ 1-\frac{Q^{\wp +2}_{p;\kappa ,\mu }(\gamma ;\alpha ,\beta ;z;m) (\phi *\Theta )(z)}{Q^{\wp +1}_{p;\kappa ,\mu }(\gamma ;\alpha ,\beta ;z;m)(\phi *\Theta )(z)}\right] \\&\quad\prec \lambda _1+\lambda _2\eta (z)+\lambda _3\,\frac{z\eta ^{\prime }(z)}{\eta (z)}, \end{aligned}$$

*then*

10 $$\left[ \frac{Q^{\wp }_{p;\kappa ,\mu }(\gamma ;\alpha ,\beta ;z;m)(\phi *\Phi )(z)}{z}\right] ^{\sigma } \left[ \frac{z}{Q^{\wp }_{p;\kappa ,\mu }(\gamma ;\alpha ,\beta ;z;m)(\phi *\Theta )(z)}\right] ^{\nu }\prec \eta (z),$$

*and*$\eta (z)$*is the best dominant*.

### *Proof*

Our aim is to apply Lemma 1. Setting

$$\rho (z)=\left[ \frac{Q^{\wp }_{p;\kappa ,\mu }(\gamma ;\alpha ,\beta ;z;m) (\phi *\Phi )(z)}{z}\right] ^{\sigma } \left[ \frac{z}{Q^{\wp }_{p;\kappa ,\mu }(\gamma ;\alpha ,\beta ;z;m) (\phi *\Theta )(z)}\right] ^{\nu }.$$

Computation shows that

$$\begin{aligned} \frac{z\rho ^{\prime }(z)}{\rho (z)}&=\lambda _3\sigma (\wp +1) \left[ \frac{Q^{\wp +1}_{p;\kappa ,\mu }(\gamma ;\alpha ,\beta ;z;m)(\phi *\Phi )(z)}{Q^{\wp }_{p;\kappa ,\mu }(\gamma ;\alpha ,\beta ;z;m)(\phi *\Phi )(z)}-1\right] \\&\quad+\nu (\wp +2)\left[ 1-\frac{Q^{\wp +2}_{p;\kappa ,\mu }(\gamma ;\alpha , \beta ;z;m)(\phi *\Theta )(z)}{Q^{\wp +1}_{p;\kappa ,\mu }(\gamma ;\alpha ,\beta ;z;m)(\phi *\Theta )(z)}\right] , \end{aligned}$$

which yields the following subordination:

$$\lambda _1+\lambda _2\rho (z)+\lambda _3\,\frac{z\rho ^{\prime }(z)}{\rho (z)}\prec \lambda _1+\lambda _2\eta (z)+\lambda _3\,\frac{z\eta ^{\prime }(z)}{\eta (z)}.$$

By setting

$$\Psi (\omega ):=\lambda _1+\lambda _2\,\omega ,\quad \Lambda (\omega ):=\frac{\lambda _3}{\omega },\,\,\,\pi \ne 0,$$

it can be easily observed that $\Psi (\omega )$ is analytic in $\mathbb {C}$ and $\Lambda (\omega )$ is analytic in $\mathbb {C}\backslash \{0\}$ and that $\Lambda (\omega )\ne 0$ when $\omega \in \mathbb {C}\backslash \{0\}$. Also, by letting

$$\begin{aligned} &\mathcal{Q}(z)=z\eta ^{\prime }(z)\Lambda (\eta (z))=\lambda _3\,\frac{z\eta ^{\prime }(z)}{\eta (z)},\\&\vartheta (z)=\Psi \left( \eta (z)\right) +\mathcal{Q}(z)=\lambda _1+\lambda _2 \eta (z)+\lambda _3\,\frac{z\eta ^{\prime }(z)}{\eta (z)}, \end{aligned}$$

we find that $\mathcal{Q}(z)$ is starlike univalent in $\mathcal{U}$ and that

$$\mathfrak {R}\left\{ \frac{z\vartheta ^{\prime }(z)}{\mathcal{Q}(z)}\right\} =\mathfrak {R}\left\{ 1+\frac{\lambda _2}{\lambda _3}\,\eta (z)+\frac{z \eta ^{\prime \prime }(z)}{\eta ^{\prime }(z)}-\frac{z\eta ^{\prime }(z)}{\eta (z)}\right\} > 0.$$

$$\square$$

### **Corollary 1**

*Let the assumptions of Theorem* 1*hold*. *Then the subordination*

$$\begin{aligned}&\lambda _1+\lambda _2\,\left[ \frac{Q^{\wp }_{p;\kappa ,\mu }(\gamma ;\alpha ,\beta ;z;m)(\phi *\Phi )(z)}{z}\right] ^{\sigma }+\lambda _3\sigma (\wp +1)\left[ \frac{Q^{\wp +1}_{p;\kappa ,\mu }(\gamma ;\alpha ,\beta ;z;m)(\phi *\Phi )(z)}{Q^{\wp }_{p;\kappa ,\mu }(\gamma ;\alpha ,\beta ;z;m)(\phi *\Phi )(z)}-1\right] \\&\quad+\prec \lambda _1+\lambda _2\eta (z)+\lambda _3\,\frac{z\eta ^{\prime }(z)}{\eta (z)}, \end{aligned}$$

*implies*

$$\left[ \frac{Q^{\wp }_{p;\kappa ,\mu }(\gamma ;\alpha ,\beta ;z;m)(\phi *\Phi )(z)}{z}\right] ^{\sigma }\prec \eta (z),$$

*and*$\eta (z)$*is the best dominant*.

### *Proof*

By letting $\nu =0$ in Theorem 1, we have the required result. $\square$

### **Corollary 2**

*Let the assumptions of Theorem* 1*hold*. *Then the subordination*

$$\begin{aligned} &\lambda _1+\lambda _2\,\left[ \frac{z}{Q^{\wp }_{p;\kappa ,\mu }(\gamma ;\alpha ,\beta ;z;m)(\phi *\Theta )(z)} \right] ^{\nu }+\nu (\wp +2)\left[ 1-\frac{Q^{\wp +2}_{p;\kappa ,\mu }(\gamma ;\alpha ,\beta ;z;m)(\phi *\Theta )(z)}{Q^{\wp +1}_{p;\kappa ,\mu }(\gamma ;\alpha ,\beta ;z;m)(\phi *\Theta )(z)}\right] \\&\quad\prec \lambda _1+\lambda _2\eta (z)+\lambda _3\,\frac{z\eta ^{\prime }(z)}{\eta (z)}, \end{aligned}$$

*implies*

$$\left[ \frac{z}{Q^{\wp }_{p;\kappa ,\mu }(\gamma ;\alpha ,\beta ;z;m) (\phi *\Theta )(z)}\right] ^{\nu }\prec \eta (z),$$

*and*$\eta (z)$*is the best dominant*.

### *Proof*

By letting $\sigma =0$ in Theorem 1, we have the required result. $\square$

### **Theorem 2**

*Let*$\eta (z)\ne 0$*be convex univalent in the open unit disk*$\mathcal{U}$. *Suppose that*

11 $$\begin{aligned} \mathfrak {R}\left\{ \frac{\lambda _2}{\lambda _3}\,\eta (z)\right\} \ge 0,\quad \nu ,\,\pi \in \mathbb {C},\,\pi \ne 0,\,\,\text{ for }\,\,z \in \mathcal{U}, \end{aligned}$$

*and that*$z\eta ^{\prime }(z)/\eta (z)$*is starlike univalent in*$\mathcal{U}$.

If $$\left[ \frac{Q^{\wp }_{p;\kappa ,\mu }(\gamma ;\alpha ,\beta ;z;m) (\phi *\Phi )(z)}{z}\right] ^{\sigma }\left[ \frac{z}{Q^{\wp }_{p;\kappa ,\mu } (\gamma ;\alpha ,\beta ;z;m)(\phi *\Theta )(z)}\right] ^{\nu }\in H[1,1]\cap \mathcal{Q}$$, where $$\Phi ,\,\Theta \in \mathcal{A},$$

$$\begin{aligned}&\lambda _1+\lambda _2\,\left[ \frac{Q^{\wp }_{p;\kappa ,\mu } (\gamma ;\alpha ,\beta ;z;m)(\phi *\Phi )(z)}{z}\right] ^{\sigma } \left[ \frac{z}{Q^{\wp }_{p;\kappa ,\mu }(\gamma ;\alpha ,\beta ;z;m)(\phi *\Theta )(z)}\right] ^{\nu }\\&\quad+\lambda _3\sigma (\wp +1)\left[ \frac{Q^{\wp +1}_{p;\kappa ,\mu }(\gamma ;\alpha ,\beta ;z;m)(\phi *\Phi )(z)}{Q^{\wp }_{p;\kappa ,\mu }(\gamma ;\alpha ,\beta ;z;m)(\phi *\Phi )(z)}-1\right] +\nu (\wp +2)\left[ 1-\frac{Q^{\wp +2}_{p; \kappa ,\mu }(\gamma ;\alpha ,\beta ;z;m)(\phi *\Theta )(z)}{Q^{\wp +1}_{p;\kappa ,\mu }(\gamma ;\alpha ,\beta ;z;m)(\phi *\Theta )(z)}\right] , \end{aligned}$$

is univalent in $\mathcal{U}$ and the subordination

$$\begin{aligned} &\lambda _1+\lambda _2\eta (z)+\lambda _3\,\frac{z\eta ^{\prime }(z)}{\eta (z)} \\&\quad\prec\lambda _1+\lambda _2\,\left[ \frac{Q^{\wp }_{p;\kappa ,\mu }(\gamma ;\alpha ,\beta ;z;m)(\phi *\Phi ) (z)}{z}\right] ^{\sigma }\left[ \frac{z}{Q^{\wp }_{p;\kappa ,\mu }(\gamma ;\alpha ,\beta ;z;m)(\phi *\Theta )(z)}\right] ^{\nu }\\&\quad+\lambda _3\sigma (\wp +1)\left[ \frac{Q^{\wp +1}_{p;\kappa ,\mu }(\gamma ;\alpha , \beta ;z;m)(\phi *\Phi )(z)}{Q^{\wp }_{p;\kappa ,\mu }(\gamma ;\alpha ,\beta ;z;m) (\phi *\Phi )(z)}-1\right] +\nu (\wp +2)\left[ 1-\frac{Q^{\wp +2}_{p;\kappa ,\mu } (\gamma ;\alpha ,\beta ;z;m)(\phi *\Theta )(z)}{Q^{\wp +1}_{p;\kappa ,\mu } (\gamma ;\alpha ,\beta ;z;m)(\phi *\Theta )(z)}\right] , \end{aligned}$$

holds, then

12 $$\eta (z)\prec \left[ \frac{Q^{\wp }_{p;\kappa ,\mu } (\gamma ;\alpha ,\beta ;z;m)(\phi *\Phi )(z)}{z}\right] ^{\sigma }\left[ \frac{z}{Q^{\wp }_{p;\kappa ,\mu } (\gamma ;\alpha ,\beta ;z;m)(\phi *\Theta )(z)}\right] ^{\nu },$$

and $\eta (z)$ is the best subordinant.

### *Proof*

Our aim is to apply Lemma 2. Setting

$$\rho (z)=\left[ \frac{Q^{\wp }_{p;\kappa ,\mu }(\gamma ;\alpha ,\beta ;z;m) (\phi *\Phi )(z)}{z}\right] ^{\sigma } \left[ \frac{z}{Q^{\wp }_{p;\kappa ,\mu }(\gamma ;\alpha ,\beta ;z;m) (\phi *\Theta )(z)}\right] ^{\nu }.$$

Computation shows that

$$\begin{aligned} \frac{z\rho ^{\prime }(z)}{\rho (z)}=\lambda _3\sigma (\wp +1)\left[ \frac{Q^{\wp +1}_{p;\kappa ,\mu }(\gamma ;\alpha ,\beta ;z;m)(\phi *\Phi )(z)}{Q^{\wp }_{p;\kappa ,\mu }(\gamma ;\alpha ,\beta ;z;m) (\phi *\Phi )(z)}-1\right] +\nu (\wp +2)\left[ 1-\frac{Q^{\wp +2}_{p;\kappa ,\mu }(\gamma ;\alpha ,\beta ;z;m)(\phi *\Theta )(z)}{Q^{\wp +1}_{p;\kappa ,\mu }(\gamma ;\alpha ,\beta ;z;m)(\phi *\Theta )(z)}\right] , \end{aligned}$$

which yields the following subordination:

$$\lambda _1+\lambda _2\eta (z)+\lambda _3\,\frac{z\eta ^{\prime }(z)}{\eta (z)}\prec \lambda _1+\lambda _2\rho (z)+\lambda _3\,\frac{z\rho ^{\prime }(z)}{\rho (z)}.$$

By setting

$$\Pi (\omega ):=\lambda _1+\lambda +2\,\omega ,\quad \Delta (\omega ):=\frac{\lambda _3}{\omega },\,\,\,\pi \ne 0,$$

it can be easily observed that $\Pi (\omega )$ is analytic in $\mathbb {C}$ and $\Delta (\omega )$ is analytic in $\mathbb {C}\backslash \{0\}$ and that $\Delta (\omega )\ne 0$ when $\omega \in \mathbb {C}\backslash \{0\}$. Also, we obtain

$$\mathfrak {R}\left\{ \frac{\Pi '\left( \eta (z)\right) }{\Delta \left( \eta (z)\right) }\right\} =\mathfrak {R}\left\{ \frac{\lambda _2}{\lambda _3}\,\eta (z)\right\} > 0.$$

Then the relation (12) follows by an application of Lemma 2. $\square$

### **Corollary 1**

*Let the assumptions of Theorem* 1*hold*. *Then the subordination*

$$\begin{aligned}&\lambda _1+\lambda _2\,\left[ \frac{Q^{\wp }_{p;\kappa ,\mu }(\gamma ;\alpha ,\beta ;z;m)(\phi *\Phi )(z)}{z}\right] ^{\sigma }+\lambda _3\sigma (\wp +1)\left[ \frac{Q^{\wp +1}_{p;\kappa ,\mu }(\gamma ;\alpha ,\beta ;z;m)(\phi *\Phi ) (z)}{Q^{\wp }_{p;\kappa ,\mu }(\gamma ;\alpha ,\beta ;z;m)(\phi *\Phi )(z)}-1\right] \\&\quad+\prec \lambda _1+\lambda _2\eta (z)+\lambda _3\,\frac{z\eta ^{\prime }(z)}{\eta (z)}, \end{aligned}$$

*implies*

$$\eta (z)\prec \left[ \frac{Q^{\wp }_{p;\kappa ,\mu }(\gamma ;\alpha ,\beta ;z;m) (\phi *\Phi )(z)}{z}\right] ^{\sigma },$$

*and*$\eta (z)$*is the best subordinant*.

### *Proof*

By letting $\nu =0$ in Theorem 1, we have the required result. $\square$

### **Corollary 3**

*Let the assumptions of Theorem* 1*hold*. *Then the subordination*

$$\begin{aligned}&\lambda _1+\lambda _2\,\left[ \frac{z}{Q^{\wp }_{p;\kappa ,\mu }(\gamma ;\alpha ,\beta ;z;m)(\phi *\Theta )(z)}\right] ^{\nu }+\nu (\wp +2)\left[ 1-\frac{Q^{\wp +2}_{p;\kappa ,\mu }(\gamma ;\alpha ,\beta ;z;m)(\phi *\Theta )(z)}{Q^{\wp +1}_{p;\kappa ,\mu } (\gamma ;\alpha ,\beta ;z;m)(\phi *\Theta )(z)}\right] \\&\quad\prec \lambda _1+\lambda _2\eta (z)+\lambda _3\,\frac{z\eta ^{\prime }(z)}{\eta (z)}, \end{aligned}$$

*implies*

$$\eta (z)\prec \left[ \frac{z}{Q^{\wp }_{p;\kappa ,\mu }(\gamma ;\alpha ,\beta ;z;m)(\phi *\Theta )(z)}\right] ^{\nu },$$

*and*$\eta (z)$*is the best subordinant*.

### *Proof*

By letting $\sigma =0$ in Theorem 1, we have the required result. $\square$

Combining Theorems 1 and 2, in order to get the following sandwich theorem.

### **Theorem 3**

*Let*$\eta _1(z)\ne 0,\,\,\eta _2(z)\ne 0$*be convex univalent in the open unit disk*$\mathcal{U},\,\lambda _i\in \mathbb {C}\,(i=1,2,3), \,\lambda _3\ne 0,\,\sigma ,\,\nu \in \mathbb {C}$*and let*$\eta _2(z)$*satisfy* (9) *and*$\eta _1(z)$*satisfy* (11) *respectively*. *Suppose that and that*$z\eta _i'(z)/\eta _i(z),\,\,i=1,2$, *is starlike univalent in*$\mathcal{U}$. *If*$\left[ \frac{Q^{\wp }_{p;\kappa ,\mu }(\gamma ;\alpha ,\beta ;z;m)(\phi *\Phi )(z)}{z}\right] ^{\sigma } \left[ \frac{z}{Q^{\wp }_{p;\kappa ,\mu }(\gamma ;\alpha ,\beta ;z;m)(\phi *\Theta )(z)}\right] ^{\nu }\in H[1,1]\cap \mathcal{Q}$, *where*$\Phi ,\,\Theta \in \mathcal{A}$,

$$\begin{aligned}&\lambda _1+\lambda _2\,\left[ \frac{Q^{\wp }_{p;\kappa ,\mu }(\gamma ; \alpha ,\beta ;z;m)(\phi *\Phi )(z)}{z}\right] ^{\sigma }\left[ \frac{z}{Q^{\wp }_{p;\kappa ,\mu }(\gamma ;\alpha ,\beta ;z;m) (\phi *\Theta )(z)}\right] ^{\nu }\\&\quad+\lambda _3\sigma (\wp +1)\left[ \frac{Q^{\wp +1}_{p;\kappa ,\mu }(\gamma ;\alpha ,\beta ;z;m)(\phi *\Phi )(z)}{Q^{\wp }_{p;\kappa ,\mu } (\gamma ;\alpha ,\beta ;z;m)(\phi *\Phi )(z)}-1\right] +\nu (\wp +2)\left[ 1-\frac{Q^{\wp +2}_{p;\kappa ,\mu }(\gamma ;\alpha ,\beta ;z;m) (\phi *\Theta )(z)}{Q^{\wp +1}_{p;\kappa ,\mu }(\gamma ;\alpha ,\beta ;z;m)(\phi *\Theta )(z)}\right] , \end{aligned}$$

*is univalent in*$\mathcal{U}$*and the subordination*

$$\begin{aligned}&\lambda _1+\lambda _2\eta _1(z)+\lambda _3\,\frac{z\eta ^{\prime }_1(z)}{\eta _1(z)} \\&\quad\prec\lambda _1+\lambda _2\,\left[ \frac{Q^{\wp }_{p;\kappa ,\mu }(\gamma ;\alpha ,\beta ;z;m)(\phi *\Phi )(z)}{z}\right] ^{\sigma }\left[ \frac{z}{Q^{\wp }_{p;\kappa ,\mu }(\gamma ;\alpha ,\beta ;z;m)(\phi *\Theta )(z)}\right] ^{\nu }\\&\quad+\lambda _3\sigma (\wp +1)\left[ \frac{Q^{\wp +1}_{p;\kappa ,\mu }(\gamma ;\alpha ,\beta ;z;m)(\phi *\Phi )(z)}{Q^{\wp }_{p;\kappa ,\mu }(\gamma ;\alpha ,\beta ;z;m)(\phi *\Phi )(z)}-1\right] +\nu (\wp +2)\left[ 1-\frac{Q^{\wp +2}_{p;\kappa ,\mu } (\gamma ;\alpha ,\beta ;z;m)(\phi *\Theta )(z)}{Q^{\wp +1}_{p;\kappa ,\mu }(\gamma ;\alpha ,\beta ;z;m)(\phi *\Theta )(z)}\right] \\&\quad\prec \lambda _1+\lambda _2\eta _2(z)+\lambda _3\,\frac{z\eta ^{\prime }_2(z)}{\eta _2(z)}, \end{aligned}$$

*holds, then*

$$\eta _1(z)\prec \left[ \frac{Q^{\wp }_{p;\kappa ,\mu }(\gamma ;\alpha ,\beta ;z;m)(\phi *\Phi )(z)}{z}\right] ^{\sigma }\left[ \frac{z}{Q^{\wp }_{p;\kappa ,\mu }(\gamma ;\alpha ,\beta ;z;m)(\phi *\Theta )(z)}\right] ^{\nu }\prec \eta _2(z),$$

*and*$\eta _1(z)$*is the best subordinant and*$\eta _2(z)$*is the best dominant*. $\square$

### **Corollary 4**

*Let the assumptions of Theorem* 3*hold and satisfy the subordination*

$$\begin{aligned}&\lambda _1+\lambda _2\frac{1+A_1z}{1+B_1z}+\lambda _3\frac{(A_1-B_1)z}{(1+A_1z)(1+B_1z)}\, \\&\quad\prec\lambda _1+\lambda _2\,\left[ \frac{Q^{\wp }_{p;\kappa ,\mu }(\gamma ;\alpha ,\beta ;z;m)(\phi *\Phi )(z)}{z}\right] ^{\sigma }\left[ \frac{z}{Q^{\wp }_{p;\kappa ,\mu }(\gamma ;\alpha ,\beta ;z;m)(\phi *\Theta )(z)}\right] ^{\nu }\\&\quad+\lambda _3\sigma (\wp +1)\left[ \frac{Q^{\wp +1}_{p;\kappa ,\mu }(\gamma ;\alpha ,\beta ;z;m)(\phi *\Phi )(z)}{Q^{\wp }_{p;\kappa ,\mu }(\gamma ;\alpha ,\beta ;z;m)(\phi *\Phi )(z)}-1\right] +\nu (\wp +2)\left[ 1-\frac{Q^{\wp +2}_{p;\kappa ,\mu } (\gamma ;\alpha ,\beta ;z;m)(\phi *\Theta )(z)}{Q^{\wp +1}_{p;\kappa ,\mu }(\gamma ;\alpha ,\beta ;z;m)(\phi *\Theta )(z)}\right] \\&\quad\prec \lambda _1+\lambda _2\frac{1+A_2z}{1+B_2z}+\lambda _3\,\frac{(A_2-B_2)z}{(1+A_2z)(1+B_2z)}, \end{aligned}$$

*Then*

$$\frac{1+A_1z}{1+B_1z}\prec \left[ \frac{Q^{\wp }_{p;\kappa ,\mu }(\gamma ;\alpha ,\beta ;z;m)(\phi *\Phi )(z)}{z} \right] ^{\sigma }\left[ \frac{z}{Q^{\wp }_{p;\kappa ,\mu }(\gamma ;\alpha ,\beta ;z;m)(\phi *\Theta )(z)}\right] ^{\nu }\prec \frac{1+A_2z}{1+B_2z},$$

*and*$\frac{1+A_1z}{1+B_1z}$*is the best subordinant and*$\frac{1+A_2z}{1+B_2z}$*is the best dominant*.

### *Proof*

Setting $\eta _1(z)=\frac{1+A_1z}{1+B_1z}\,(-1\le B_1<A_1\le 1)$ and $\eta _2(z)=\frac{1+A_2z}{1+B_2z}\,(-1\le B_2 <A_2\le 1)$ in Theorem 3. $\square$

## Conclusion

By the term of the extend fractional hypergeometric function, we defined a new fractional integral operator in the open unit disk. This operator is a generalization of the well known Noor integral operator. Based on this operator, many subclasses may introduce in the geometric function theory. In this study, we concerned with a specific class of analytic function called of convolution type. This class imposed several well known classes.
